# Psychotropic drug purchases during the COVID-19 pandemic in Italy and their relationship with mobility restrictions

**DOI:** 10.1038/s41598-022-22085-4

**Published:** 2022-11-11

**Authors:** Francesca Marazzi, Andrea Piano Mortari, Federico Belotti, Giuseppe Carrà, Ciro Cattuto, Joanna Kopinska, Daniela Paolotti, Vincenzo Atella

**Affiliations:** 1 Centre for Economic and International Studies, University of Rome Tor Vergata, 00133 Rome, Italy; 2 Department of Economics and Finance, University of Rome Tor Vergata, 00133 Rome, Italy; 3 School of Medicine and Surgery, University of Milano Bicocca, 20126 Milan, Italy; 4 ISI Foundation, 10126 Turin, Italy; 5 Department of Informatics, University of Turin, 10124 Turin, Italy; 6 Department of Social Sciences and Economics, University of Rome La Sapienza, 00185 Rome, Italy; 7 Directorate General for Planning, Ministry of Health, 00144 Rome, Italy

**Keywords:** Psychology, Anxiety, Depression, Health care economics

## Abstract

Recent literature on the mental health consequences of social distancing measures has found a substantial increase in self-reported sleep disorders, anxiety and depressive symptoms during lockdown periods. We investigate this issue with data on monthly purchases of psychotropic drugs from the universe of Italian pharmacies during the first wave of the COVID-19 pandemic and find that purchases of mental health-related drugs have increased with respect to 2019. However, the excess volumes do not match the massive increase in anxiety and depressive disorders found in survey-based studies. We also study the interplay between mobility, measured with anonymized mobile phone data, and mental health and report no significant effect of mobility restrictions on antidepressants and anxiolytics purchases during 2020. We provide three potential mechanisms that could drive the discrepancy between self-reported mental health surveys and psychotropic drugs prescription registries: (1) stockpiling practices in the early phases of the pandemic; (2) the adoption of compensatory behavior and (3) unexpressed and unmet needs due to both demand- and supply-side shortages in healthcare services.

The COVID-19 epidemic, caused by the novel coronavirus SARS-COV-2, originated in Wuhan, China, at the end of 2019 and spread rapidly worldwide in the following months. In the absence of effective treatments to prevent the contagion, countries have implemented diverse Non-Pharmaceutical Interventions (NPIs)^1,2^. School closures, travel restrictions, national lockdowns and various social distancing measures affected people’s lives from different perspectives.

Italy was the first Western country to experience a significant and rapid outbreak of the virus, with the first infected patient isolated on February 18th 2020. As early as of March 9th 2020, Italy declared a complete national lockdown which lasted until May 18th 2020 (the so-called “phase 1”), with other severe mobility restrictions that continued until June 3rd (“phase 2”). In parallel, the World Health Organization declared COVID-19 a global pandemic on March 11th 2020, followed by large-scale NPIs introduced in many other countries. The events of the following months caused a widespread and dramatic disruption in daily lives, with an emotional, social and economic burden that still remains to be understood^3–5^.

The primary goal of this paper is to evaluate, in the Italian context, the impact of the nationwide NPIs and restrictions on mental health issues among the general population through the assessment of purchases of mental health-related drugs at the national level.

According to the OECD^6^, the world prevalence of mental health problems was on a stable path until the outbreak of COVID-19 in 2020, when rates of depressive and anxiety disorders increased in several countries. In particular, self-reported prevalence has more than doubled in Belgium, France, Italy, Mexico, New Zealand, the United Kingdom and the United States in the case of anxiety; and in Australia, Belgium, Canada, France, the Czech Republic, Mexico, Sweden, the United Kingdom and the United States in the case of depression. Despite limited representativeness and cross-country comparability of these surveys due to different designs and sampling techniques, the evidence clearly points to a significant increase in the prevalence of subjective anxiety and depressive disorders.

A recent WHO report provides a comprehensive overview of the mental health effects of the COVID-19 pandemic^7^, based on the Global Burden of Disease data and other sources^3^, including systematic reviews and meta-analyses^8–10^. According to the GBD^3^, the pandemic has led to a 27.6% increase in major depressive disorder (MDD) cases and a 25.6% increase in anxiety disorders (AD) cases worldwide in 2020. Overall, the pandemic is estimated to have caused 137.1 additional disability-adjusted life years (DALYs) per 100,000 population for MDD and 116.1 for AD. The report suggests that the surge in mental health disorders has disproportionately affected the young, females and patients with pre-existing health conditions.

Results based on longitudinal data and mental health trajectories are more optimistic: studies based on a weekly survey in the UK find a sharp increase in depression and anxiety symptoms at the beginning of the lockdown^11–13^, followed by a common declining trajectory in the weeks after the introduction of mobility restrictions. The authors also highlight how individuals with previous diagnoses of mental health conditions reported higher anxiety and depression in the early phases of the lockdown compared to the rest of the sample, but did not show higher levels of emotional reactivity, possibly due to their experience with coping strategies in stressful situations.

Overall, despite the vast proliferation of studies that relate COVID-19 to population mental health, the evidence does not yield convergent nor robust results on the phenomenon. To the best of our knowledge, to date there is a limited number of studies based on objective data^14–17^.

Concerning the Italian case, many studies documenting mental health effects of COVID-19 are based on online surveys and self-reported mental health status and several of those suffer from limited representativeness of the data and/or small sample sizes^18–20^. Overall, these studies find a consistent deterioration of the mental health status of Italians^21,22^, in particular for females, the young and patients with pre-existing conditions and worse socio-economic status^23,24^. The prevalences of (self-reported) anxiety and depressive symptoms observed in the cited studies, which range between 17.6 and 41.5% for the first and between 12.4 and 33.2% for the second, are strikingly above the prevalence of depressive symptoms reported by the Italian National Health Institute (ISS) for the pre-pandemic period (6% for 2016–2019).

Gualano et al.^25^ note that these studies report a greater prevalence of depressive and anxiety symptoms in the Italian population during the lockdown, despite a reduction in voluntary admissions in the 40 days after the beginning of COVID-19 epidemic in Italy. Furthermore, the authors highlight a non-significant increase in outpatient pharmaceutical consumption of antidepressants before (January and February 2020) and after (March and April 2020) lockdown, although they evidence a significant one for anxiolytics^26^. More recent works confirm that while women and younger individuals were found to be particularly prone to the risk of depressive symptoms, as a result of the pandemic, the difference in the prevalence of depressive symptoms before and after the first lockdown is not statistically significant^27,28^.

Interestingly, the Italian Observatory of Drug Consumption (OSMED), led by the Italian Medicines Agency (AIFA), documents a significant increase in the use of anxiolytics and sedatives over the last years due to a recent surge in stress disorders^29^. In Italy, Benzodiazepine consuption raised from 47.4 DDD/1000 inhabitants-die in 2014 to 55.0 in 2020, while that of antidepressants from 39.2 in 2014 to 43.6 in 2020. According to AIFA, this rise is symptomatic of a consolidated habit of resorting easily to pharmaceutical therapies, urging more controlled prescribing practices. Additionally, the same report mentions an increase in the prevalence of depression in the latest years, with a consequent increase in GPs’ attention and management skills, reducing the need to employ pharmacological treatments to only 1 out of 3 patients with depressive symptoms.

Our aim in this context is to provide a measurable perspective of the mental health consequences of COVID-19 in Italy during the first year of the pandemic. We use nationwide detailed data for the purchases of anxiolytics and antidepressants (measured at three levels of Anatomical Therapeutic Chemical (ATC) codes) to evaluate the variation in the consumption of anxiolytics and antidepressants in Italy in the early months of 2020 with respect to 2019. Additionally, we study the correlation of drug purchase patterns with high-resolution mobile phones mobility data within and across Italian provinces. Using a multivariate regression approach, we test whether there is an interplay between the consumption of drugs for mental health disorders and restrictions on individuals’ mobility, controlling for the geographical heterogeneity of the intervention measures, COVID contagion and mortality. To this aim, we measure two different kinds of mobility flows, namely the one between home and workplace versus the one between home and other locations. By limiting our analysis to the first year of the COVID-19 pandemic, we avoid potential confounding effects of long-covid on mental health, given that “severe acute COVID-19 illness—indicated by extended time bedridden—is associated with long-term mental morbidity among recovering individuals in the general population”^30^.

We find that purchases of mental health-related drugs have increased with respect to 2019, but the excess volumes do not match the massive increase in anxiety and depressive disorders found in survey-based studies. Furthermore, while we find incremental effects on anxiolytics consumption in the months corresponding to the national lockdown introduction, we do not observe any further significant effect of mobility restrictions. We interpret the divergence of our results with respect to findings based on self-administered surveys according to three main hypotheses. First, the mismatch is likely coherent with a milder nature of self-reported psychological distress with respect to conditions requiring pharmaceutical interventions. Second, as we observe an increase in anxiolytics and antidepressants consumption in the last part of 2020, we conclude that a consistent share of mental health disorder cases might have been overlooked during the first year of the pandemic, possibly leading to the onset of more severe conditions in a longer term. Third, individuals affected by the pandemic distress might have exhibited differential mental health responses due to their differential (optimal) investments in defensive expenditures, coping mechanisms or compensatory behaviors, such as mild non-specific drugs, support groups or specialist psychotherapy. Our estimates of the marginal effect of the COVID-19 pandemic on mental health are thus likely to measure the net effect, whose welfare and policy implications highlight an essential role for economic incentives determining such defensive spending.

## Results

**Figure 1:**
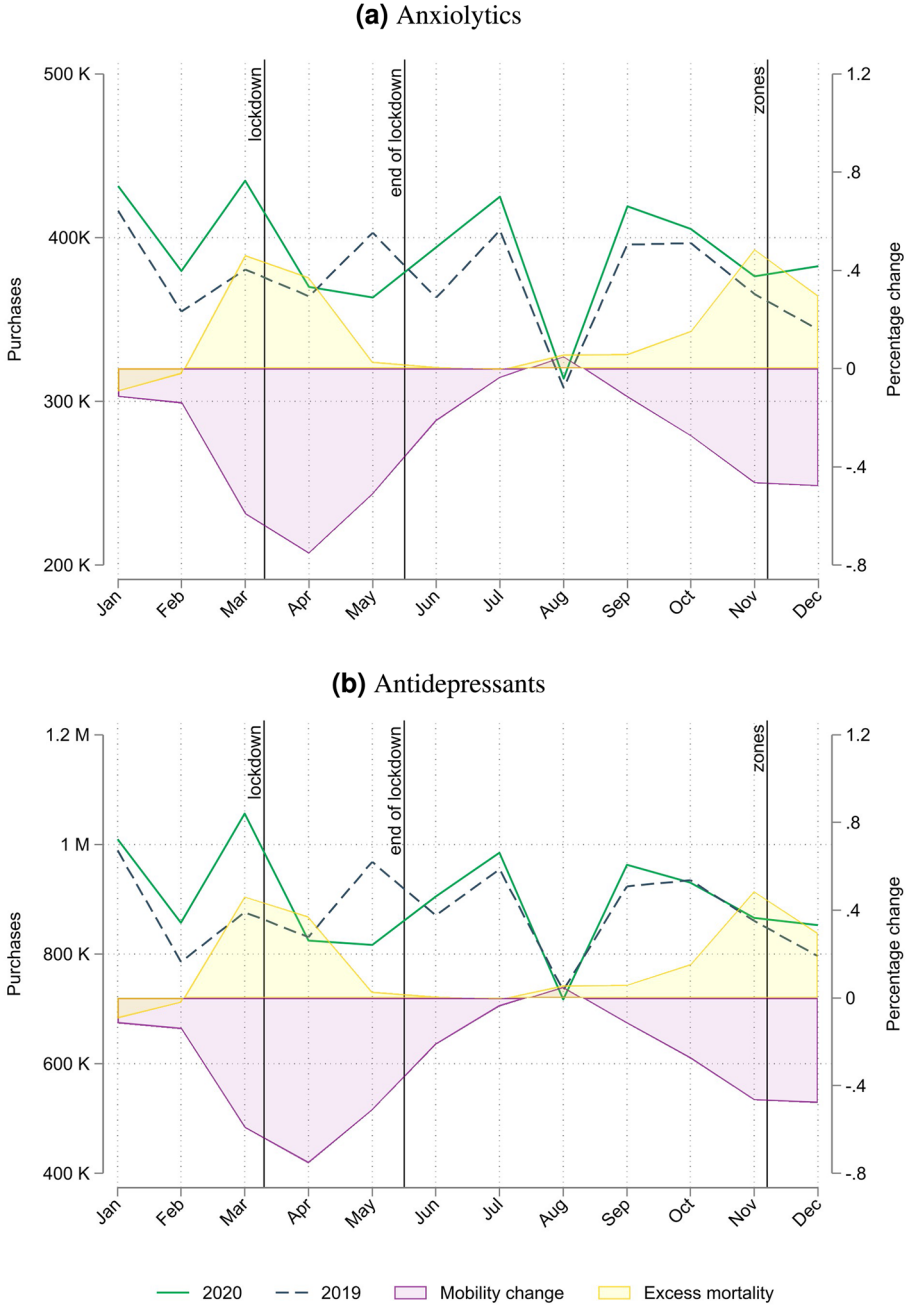
Monthly purchases of anxiolytics and antidepressants in standard units for 2020 and 2019. Average municipality-level drugstores’ purchases in DDD per month, for 2020 (green solid line) and 2019 (blue dashed line) of anxiolytics (panel **a**, ATC code N05B) and antidepressants (panel **b**, ATC code N06A). The yellow shaded area reports monthly excess death in percentage change for 2020 with respect to the average number of deaths in the period 2015–2019, while the purple shaded area reports the average change in mobility with respect to mobility patterns in January 2020 (see “Methods” section for a detailed description of the mobility algorithm used). Vertical lines indicate the beginning and end of the national lockdown and the creation of zones with differentiated mobility restrictions.  Source: our calculation on IQVIA, Teralytics and ISTA﻿T data.

Figure 1 traces the evolution of anxiolytics and antidepressants purchases, respectively, together with the most salient moments of the COVID-19 pandemic in Italy. The vertical lines mark the most stringent containment measures, i.e., the national lockdown (from March 9th to May 18th) and the institution of ‘zones’ with differentiated mobility restrictions on November 6th. The background of the two Figures additionally shows the evolution of the monthly excess mortality (yellow area) and the change in mobility (purple area) in 2020. Most importantly, the figures picture monthly drugstore purchases for 2019 (dashed blue line) and 2020 (solid green line).

We detect clear seasonality patterns for both anxiolytics (Fig. 1a) and antidepressants (Fig. 1b). The differences in each month-of-year purchases are not very large, although, with the exception of April and March, the volumes in 2020 supersede the ones in 2019. In March 2020, the onset of the national lockdown, we observe a pronounced spike in the purchases of both classes of drugs, followed by a substantial drop in the subsequent 2 months. In fact, in May 2020, at the end of the national lockdown, the purchases are significantly lower compared to 2019. During the summer months, the differentials in purchases shrink and start to widen again only in November for antidepressants and in December 2020 for anxiolytics.

Figures A.1a,b in the Appendix show the evolution of the percentage change in both drugstore purchases and reimbursable prescriptions in 2020, with respect to 2019. Indeed, in the case of antidepressants, drugstore purchases mirror customer purchases dispensed upon reimbursable prescription, confirming that the share of antidepressants sold without prescription is negligible. Conversely, there seems to be a systematic proportion of anxiolytics dispensed without medical prescription. Table A.2 in the Appendix shows that, overall, the 2020 vs. 2019 differences in monthly purchases are statistically significant for both anxiolytics (panel *A*) and antidepressants (panel *B*).

**Table 1:** Anxiolytics, percent variation.

	Model 1	Model 2	Model 3	Model 4	Model 5	Model 6
March–Dec	0.0002 (0.0101)					
Mar		0.0822*** (0.0100)	0.0715*** (0.0125)	0.0656 (0.0371)	0.0717*** (0.0116)	0.0762*** (0.0148)
Apr		$-$ 0.0441*** (0.0121)	$-$ 0.0624*** (0.0147)	0.0107 (0.0870)	$-$ 0.0654*** (0.0153)	$-$ 0.0247 (0.0352)
May		$-$ 0.1457*** (0.0096)	$-$ 0.1528*** (0.0115)	$-$ 0.1338*** (0.0233)	$-$ 0.1519*** (0.0112)	$-$ 0.1592*** (0.0140)
Jun		0.0511*** (0.0108)	0.0498*** (0.0111)	0.0416*** (0.0115)	0.0469*** (0.0118)	0.0409** (0.0152)
Jul		0.0124 (0.0108)	0.0121 (0.0108)	0.0062 (0.0109)	0.0070 (0.0110)	0.0064 (0.0137)
Aug		$-$ 0.0282* (0.0109)	$-$ 0.0222 (0.0117)	$-$ 0.0290* (0.0121)	$-$ 0.0331** (0.0124)	$-$ 0.0421* (0.0173)
Sep		0.0247* (0.0120)	0.0232 (0.0121)	0.0208 (0.0133)	0.0186 (0.0123)	0.0195 (0.0158)
Oct		$-$ 0.0064 (0.0121)	$-$ 0.0098 (0.0125)	$-$ 0.0134 (0.0142)	$-$ 0.0105 (0.0124)	$-$ 0.0141 (0.0152)
Nov		0.0024 (0.0115)	$-$ 0.0061 (0.0126)	0.0082 (0.0194)	$-$ 0.0063 (0.0121)	$-$ 0.0040 (0.0167)
Dec		0.0933*** (0.0195)	0.0834*** (0.0199)	0.1134*** (0.0280)	0.0802*** (0.0209)	0.0855** (0.0260)
Mobility			$-$ 0.0225 (0.0134)	0.0160 (0.0302)		
Mobility (home to work)					$-$ 0.0352 (0.0244)	$-$ 0.0931 (0.0525)
Mobility (Home to other)					$-$ 0.0003 (0.0225)	0.0698 (0.0506)
Excess mortality $\times$ 1k inhab, last month	$-$ 0.0611*** (0.0139)	$-$ 0.0050 (0.0066)	$-$ 0.0035 (0.0065)	$-$ 0.0020 (0.0066)	$-$ 0.0033 (0.0065)	$-$ 0.0025 (0.0067)
Contagions $\times$ 1k inhab, last month	0.0002*** (0.0000)	$-$ 0.0000 (0.0000)	$-$ 0.0000 (0.0000)	$-$ 0.0000 (0.0000)	$-$ 0.0000 (0.0000)	$-$ 0.0000 (0.0000)
Region specific time trend	Yes	Yes	Yes	Yes	Yes	Yes
Mobility $\times$ months	No	No	No	Yes	No	Yes
N. Obs	1177	1177	1144	1144	1144	1144

Robust standard errors in parentheses, * for p < 0.05, ** for p < 0.01, and *** for p < 0.001. The dependent variable is the percent variation with respect to 2019 of the monthly DDD of anxiolytics purchased by Italian pharmacies at the Province level. February is the reference category. Coefficients for monthly dummies are increase or decrease with respect to the percent variation of purchases in February.

Tables 1 and 2 report regression results for 6 different model specifications for anxiolytics and antidepressants purchases, respectively. The most parsimonious specification (Model 1) includes a dummy variable that captures the overall effect of the pandemic period (March–December 2020) as well as the intensity of contagion and excess mortality. The coefficient estimate on the local excess mortality is negative, while the one on contagion is positive, both being statistically significant.

In order to account for the heterogeneity in the impact of the pandemic over time, we introduce month-specific time dummies (Model 2), which wipe out the effects of both excess mortality and contagions. The sign and magnitude of the coefficients of the month dummies are consistent with the patterns observed in Fig. 1a: a positive and significant increase in the early stages of the lockdown, followed by a reduction in the subsequent months and a sharp increase in December. While the time dummies effectively capture the evolution of the pandemic at the national level, one might argue that they do not explain the heterogeneity in the differentials of how single territories were affected by lockdowns. For this reason, we further enrich our specification with the aggregate indicator of monthly changes in mobility (Model 3). However, mobility fluctuations do not seem to affect purchases of anxiolytics. Nor do we find any effect of mobility in Model 4, when it is interacted with month-specific dummies, or when, in models 5 and 6, we distinguish home-to-work from home-to-other mobility type (the interaction coefficients are never statistically significant and are not reported in the tables, but are available upon request). The multitude of model specifications that we adopt shows that net of scattered spikes in purchases in March, June and December, different local mobility patterns play no role in determining the utilization of psychotropic drug patterns.

The results for antidepressants are very similar: mobility, the number of contagions and excess mortality do not seem to play any role, while the time dummies show larger magnitudes which are all strongly significant, with the exception of the month of December.

**Table 2:** Antidepressants, percent variation.

	Model 1	Model 2	Model 3	Model 4	Model 5	Model 6
March–Dec	$-$ 0.0175** (0.0067)					
Mar		0.1234*** (0.0068)	0.1229*** (0.0081)	0.1075*** (0.0298)	0.1218*** (0.0077)	0.1100*** (0.0121)
Apr		$-$ 0.0902*** (0.0070)	$-$ 0.0956*** (0.0094)	$-$ 0.0213 (0.0579)	$-$ 0.0981*** (0.0092)	$-$ 0.0832*** (0.0193)
May		$-$ 0.2320*** (0.0072)	$-$ 0.2326*** (0.0081)	$-$ 0.2352*** (0.0158)	$-$ 0.2332*** (0.0077)	$-$ 0.2426*** (0.0102)
Jun		$-$ 0.0339*** (0.0077)	$-$ 0.0346*** (0.0079)	$-$ 0.0436*** (0.0080)	$-$ 0.0355*** (0.0083)	$-$ 0.0441*** (0.0098)
Jul		$-$ 0.0464*** (0.0078)	$-$ 0.0466*** (0.0079)	$-$ 0.0487*** (0.0079)	$-$ 0.0478*** (0.0085)	$-$ 0.0502*** (0.0108)
Aug		$-$ 0.0975*** (0.0095)	$-$ 0.0946*** (0.0096)	$-$ 0.0966*** (0.0095)	$-$ 0.0972*** (0.0104)	$-$ 0.0972*** (0.0135)
Sep		$-$ 0.0270* (0.0103)	$-$ 0.0275** (0.0103)	$-$ 0.0310** (0.0101)	$-$ 0.0287** (0.0107)	$-$ 0.0384** (0.0126)
Oct		$-$ 0.0732*** (0.0117)	$-$ 0.0737*** (0.0118)	$-$ 0.0729*** (0.0123)	$-$ 0.0742*** (0.0118)	$-$ 0.0745*** (0.0131)
Nov		$-$ 0.0615*** (0.0125)	$-$ 0.0630*** (0.0130)	$-$ 0.0847*** (0.0148)	$-$ 0.0638*** (0.0129)	$-$ 0.0663*** (0.0146)
Dec		0.0050 (0.0169)	0.0013 (0.0171)	0.0098 (0.0214)	$-$ 0.0004 (0.0171)	$-$ 0.0013 (0.0185)
Mobility			$-$ 0.0043 (0.0087)	0.0194 (0.0186)		
Mobility (home to work)					$-$ 0.0100 (0.0160)	$-$ 0.0342 (0.0297)
Mobility (home to other)					$-$ 0.0007 (0.0170)	0.0464 (0.0343)
Excess mortality $\times$ 1k inhab, last month	$-$ 0.0768*** (0.0168)	$-$ 0.0034 (0.0043)	$-$ 0.0024 (0.0043)	$-$ 0.0010 (0.0044)	$-$ 0.0022 (0.0044)	$-$ 0.0019 (0.0045)
Contagions $\times$ 1k inhab, last month	0.0002*** (0.0000)	0.0000 (0.0000)	0.0000 (0.0000)	0.0000 (0.0000)	0.0000 (0.0000)	0.0000 (0.0000)
Region specific time trend	Yes	Yes	Yes	Yes	Yes	Yes
Mobility $\times$ months	No	No	No	Yes	No	Yes
N. Obs	1177	1177	1144	1144	1144	1144

Robust standard errors in parentheses, * for p < 0.05, ** for p < 0.01, and *** for p < 0.001. The dependent variable is the percent variation with respect to 2019 of the monthly DDD of antidepressants purchased by Italian pharmacies at the Province level. February is the reference category. Coefficients for monthly dummies are increase or decrease with respect to the percent variation of purchases in February.

## Discussion

In Italy, the market for antidepressants is almost double compared to anxiolytics (see Table A.1 in the Appendix), which is similar to Portugal^31^. In terms of dispensing, anxiolytics are mostly sold without physician prescription ($92.73\%$ in 2019 and $93.01\%$ in 2020), while antidepressants are mainly dispensed with prescription ($85.65\%$ in 2019 and $85.49\%$ in 2020). Given these different dispensing practices, in the analysis we focus on overall volumes of drugstores’ purchases of anxiolytics and antidepressants separately, rather than considering the single channels of dispensing.

The increase in purchases of anxiolytics and antidepressants during 2020 was only moderate compared to 2019 and mainly concentrated in the early months of the pandemic (February and March). The coefficient estimate on the local excess mortality is negative, while the one on contagion is positive, both being statistically significant. On the one hand, psychotropic drugs consumption represents concomitant therapies in neuro-degenerative disorder treatments and in the elderly in general^32,33^, and given that COVID-19 excess mortality was disproportionately higher among the elderly, the latter is likely to drive a decrease in consumption. On the other hand, higher contagion rates are likely to promote suffering and distress, which, net of lockdown measures, is expected to promote a surge in anxiety disorders. Furthermore, we show that net of the national lockdown effect, region-specific trends in purchases in 2020, and the evolution of the virus spread in terms of contagion and mortality, there is no association between psychotropic drug volumes and changes in mobility.

If mobility patterns effectively capture the heterogeneities in effective confinement and social distancing measures, the evidence of our analysis diverges from the prevailing findings in the literature, which point to a pronounced surge in the prevalence of anxiety and depression disorders^21^.

On the other hand, our findings are consistent with the only study based on a large nationally representative sample of individuals from the general adult population residing in Italy comparing mental distress before and after the pandemic^28^. The authors highlight that “the increase of depressive symptoms observed during the pandemic was small in size and as such of limited meaningfulness from a clinical point of view”.

Below, we present and discuss possible mechanisms explaining why the surge in mental distress reported in studies carried out during the lockdown is not reflected in psychotropic drug purchases.

**Figure 2:**
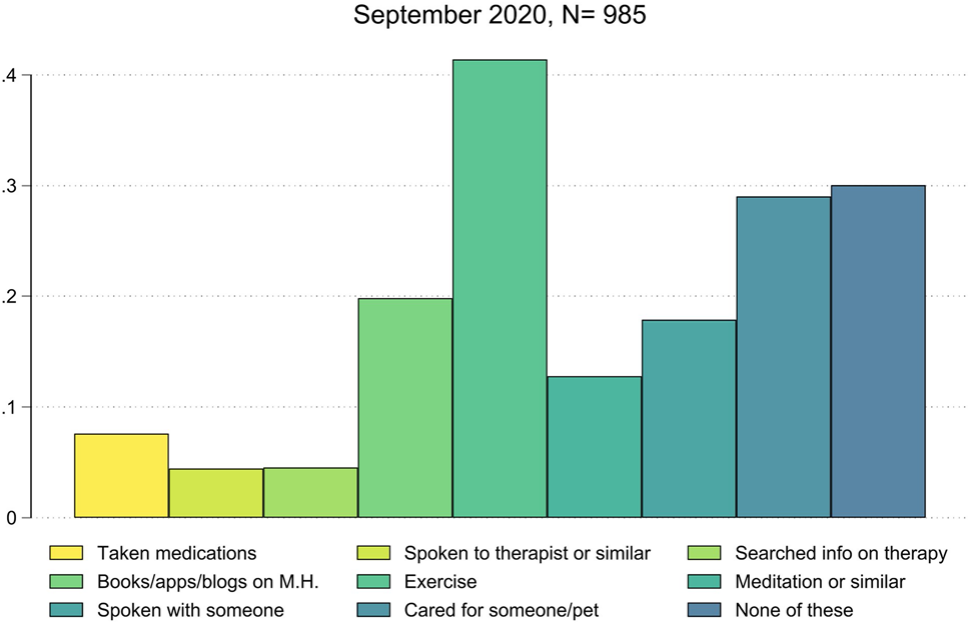
Self-reported solutions for stress, mental health and mental wellbeing (Italian representative sample). Answers to the question: “In the past week have you done any of the following [to improve your stress, mental health or mental wellbeing]? Please tick all that apply”. Source: Our elaboration of data provided by Imperial College London YouGov COVID-19 Behaviour Tracker Data Hub^39^.

- *Stockpiling*. The trends in purchases we observe are in line with the stockpiling practices already documented by other studies during 2020, with consequences at different levels. On the one hand, if stockpiling behavior is adopted by healthcare providers, as was the case with U.S. hospitals in the early phases of the pandemic, it may lead to short-run shortages^34^. On the other hand, if adopted by consumers, stockpiling or “panic buying” with the scope of preventive consumption smoothing is likely to cause uneven purchase patterns over time, as evidenced during the first COVID-19 wave in several product categories, including medicines^35^, and antidepressants in particular^36^. This last practice seems to be more in line with what we document for March 2020, when we observe a pronounced peak of purchases, followed by a proportional reduction in April and May. This implies that a large portion of the increase in the quantity *stocked* did not reflect an equivalent real increase in the quantity *demanded*. As a matter of fact, purchases in summer 2020 and during the second wave exceed only slightly those of 2019.
- *Compensatory behavior and defensive expenditure*. Adopting compensatory behavior before engaging in psychiatric therapies might also be a reason for the mismatch between the perceived disruption in mental health conditions and drug consumption. A number of these practices have become easily accessible online, with recent studies documenting mental health benefits accruing from mindfulness meditation apps^37^, and an increasing proportion of psychotherapy sessions delivered online during the lockdowns^38^. The hypothesis of engaging in compensatory behavior during 2020 is also supported by the results of the Imperial College London on the adoption of a broad set of solutions to reduce stress and improve mental health and wellbeing^39^. Focusing on the subsample of Italians only, Fig. 2 shows that they frequently adopted self-care practices (e.g., physical exercise and meditation) and social connections, while resorting less frequently to medications and even less to mental health professionals. Interestingly, the results for Italy are in line with the rest of Europe (Figure A.2 in the Appendix plots the respective evidence for other European countries). It is then clear that these behaviors moderate the effect of COVID-19 mobility restrictions on mental health, implying an underestimation of the actual effect. From a policy perspective and welfare implications, it is thus crucial to account for both the benefits (lower drug expenditure) and the costs of compensatory behavior (non-pharmaceutical and non-medical therapies), as there may be an important scope for economic incentives promoting such investments.
- *Unexpressed and unmet needs*. Unexpressed needs originate from the demand side and are usually defined as unexpressed demand due to either a cultural reluctance in resorting to mental health professionals or very mild conditions that do not require pharmaceutical and medical therapies. On the contrary, unmet needs signal supply side problems. For example, it is possible that during lockdowns patients may have either lost access to adequate mental health care (if already on therapy) or had difficulties accessing it (if new patients)^40,41^, given also the considerable difficulties the whole healthcare system was experiencing^42^. While there is no clear evidence in favor or against the first hypothesis, Figure A.1 in the Appendix supports the second hypothesis. The Figure shows the year-over-year monthly percentage change between 2019 and 2020 of overall drugstore purchases and reimbursed prescription-only drug purchases for anxiolytics and antidepressants. In particular, Figure A.1a suggests that the percentage change of reimbursable anxiolytics has been, except for March, below their 2019 levels. This stylized fact points to difficulty obtaining (reimbursable) drugs, that most frequently are prescribed by GPs or specialists^43^. In fact, drugstores’ overall purchases have been consistently above their 2019 levels, suggesting that patients may have partially overcome the problem by resorting to anxiolytics that do not require a GP or a specialist prescription (and may also signal mild mental health conditions). This behavior is also coherent with the above “panic buying” pattern observed in March, followed by a sharp decrease, also in prescribed quantities. At the same time, we can safely rule out any stockpiling by the pharmacies, as they strongly rely on just-in-time management of their purchases.
  Antidepressants data in Figure A.1b further support this hypothesis. Year-over-year monthly percentage changes between 2019 and 2020 of drugstores’ overall purchases and reimbursable prescription drugs followed similar patterns without any relevant mismatch. By comparing the rates of changes in both markets and given the different prescription rules for anxiolytics and antidepressants, we can infer that patients with diagnosed chronic conditions (i.e., depression) might have encountered fewer obstacles in obtaining prescriptions to continue their therapies smoothly. In contrast, new incident patients with non-chronic or episodic disorders (such as anxiety) may have either experienced more difficulties accessing adequate mental healthcare services or not considered it at all (see the point on compensatory behavior), in line with the hypothesis of unmet needs.

Finally, although a surge in the prevalence of mental health conditions did not happen, it is worthwhile to stress that we do observe a consistent increase in purchases of anxiolytics and antidepressants in December 2020 with respect to 2019. If our interpretations of the phenomenon are correct, it is well possible that numerous new cases of mental illness have been overlooked and not yet targeted by formal care, either because patients did not express their needs and resorted to alternative solutions, or because the healthcare system was unable to intercept them. This evidence pushes to an even higher attention to mental health for the years to come, especially for high risk groups such as women and individuals with financial difficulties^28^.

## Methods

In order to uncover the interplay between mental health and the COVID-19 pandemic, we employ a fixed-effects regression analysis based on observational drug purchases and mobility data.

### Data

#### Drugs

Our analysis is based on monthly purchases of psychotropic drugs of the universe of Italian pharmacies from January 1st 2019 to December 31st 2020, which are then aggregated at the administrative level in 107 provinces belonging to 20 regions. Data on anxiolytics and antidepressants are provided by IQVIA (https://www.iqvia.com) and consist of anonymized data on the number of Defined Daily Doses (DDDs) purchased by Italian drugstores at the municipality level by ATC code (3 digits) and the amount sold via reimbursable prescriptions. For the purposes of this paper, we focus on drugs belonging to ATC codes N05B (anxiolytics) and N06A (antidepressants). The DDDs not sold via Reimbursable Prescriptions (R-Rx) is either sold via Non-Reimbursable Prescriptions (NR-Rx), over the counter, or kept as pharmacies’ stock. However, given that for DDDs not sold via R-Rx we cannot observe the specific selling channel, we focus our analysis mainly on overall drugstore purchases. We focus on overall volumes of drugstores’ purchases of anxiolytics and antidepressants separately, rather than considering the single channels of dispensing. Additional details concerning the volume of DDD sold via reimbursable prescriptions are provided in the Appendix.

#### Mobility

We use mobility data collected by Teralytics (https://www.teralytics.net), which gathers individual anonymized data from mobile phones for the providers Wind and 3 (approximately 30% of the Italian population). Teralytics provides the daily number of trips aggregated at the sub-Province level (between LAU1, i.e. NUTS3, and LAU2) in a dyadic form, from January 1st to December 31st 2020. Our data distinguishes trips according to the means of transportation (road, train, plane and not classified) and the reason for movement. In particular, we can identify the individual’s place of residence as the place where (s)he regularly spends the night and subsequently labels trips according to their purpose, *home-to-work, home-to-other, etc.*. Data for the Friuli-Venezia Giulia region are provided at the sub-regional level and the unique Province for which data are identifiable is Trieste, the Regional County Seat. Therefore, mobility data are missing for the Provinces of Udine, Gorizia and Pordenone.

Let us define the number of trips from location *l*, $l = (1,\ldots ,L)$, on day of week *d*, $d = (1,\ldots ,7)$, and with purpose of trip *p*, $p \in$*{home-to-work, home-to-other, work-to-home, work-to-other, other-to-home, other-to-work, others}*, as $V_{l,d,p}$. We then define the baseline $B_{l,d,p}$ as the average of $V_{l,d,p}$ in all weeks of January 2020 (e.g. the average number of home-to-work trips from the Province of Milan to any other Province on all Mondays of January). The change in purpose-specific mobility with respect to the baseline is then defined as

$$\begin{aligned} V'_{l,d,p} = \frac{V_{l,d,p} - B_{l,d,p}}{B_{l,d,p}}, \end{aligned}$$

while we compute the ‘Overall’ mobility change as

$$\begin{aligned} V'_{l,d} = \frac{\sum _p V_{l,d,p} - \sum _p B_{l,d,p}}{\sum _p B_{l,d,p}}. \end{aligned}$$

In order to have a common aggregation level across pharmaceutical and mobility data, we average daily mobility changes $V'_{l,d,p}$ and $V'_{l,d}$ at the month and Province (NUTS3) level.

#### Additional controls

Data on daily all-causes deaths and municipalities’ population are provided by the Italian National Statistical Institute (ISTAT). We construct an indicator of excess mortality as the percent variation of the number of monthly excess deaths in each month of 2020 with respect to the average monthly number of deaths for the period 2015–2019. Data on daily COVID-19 cases are provided by the Italian Government, Department of Civil Protection and are used to compute a measure of per-capita infections at month and province level.

### Regression analysis

The outcome studied is the monthly percent variation of purchased DDDs of anxiolytics and antidepressants with respect to the same month in 2019 at province level. In order to investigate the relationship between these outcomes and the mobility variation compared to a pre-pandemic situation, we used the following province level two-way fixed-effects model:

1 $$\begin{aligned} y_{it} = \beta m_{it} + \gamma _t m_{it} d_{it} + {\mathbf {z}}_{it-1}\varvec{\theta } + \mu _j d_j t + c_i + \tau _t + \epsilon _{it} \qquad t= 3, \ldots , 12 \end{aligned}$$

where $m_{it}$ is our mobility indicator for province *i* and month *t*, $d_{it}$ a dummy for the *t*-th month of 2020 (base category February), $\mathbf{z} _{it-1}$ includes both the one-period lagged excess mortality, as a proxy for the threat of mortality, and, to measure the effect of contagion, the number of COVID-19 cases per capita, $d_j t$ is a linear time trend for region *j*, with $d_j$ being dummy for the *j*-th region, $c_i$ is the province specific fixed-effect to purge the time invariant province specific unobservables, $\tau _t$ controls for aggregate month effects, and $\epsilon _{it}$ is the usual idiosyncratic error term.

## Supplementary Information

Supplementary Information.

## Data Availability

The data that support the findings of this study are available from IQVIA (drug purchases) and Teralytics (mobility data) but restrictions apply to the availability of these data, which were used under license for the current study, and so are not publicly available. Data are however available from Vincenzo Atella (atella@uniroma2.it) upon reasonable request and with permission of IQVIA (drug purchases) and Teralytics (mobility data). Data on daily all-causes deaths are available in the repository of the Italian National Statistical Institute (https://www.istat.it/it/archivio/240401). Data on daily COVID-19 cases are available in the repository of the Italian Government, Department of Civil Protection (https://github.com/pcm-dpc/COVID-19).
