# Supplementary material for: Psychotropic drug purchases during the COVID-19 pandemic in Italy and their relationship with mobility restrictions
Source: Sci Rep. 2022 Nov 11;12:19336. doi: 10.1038/s41598-022-22085-4 (PMC9651906; doi:10.1038/s41598-022-22085-4)
Supplement: Supplementary file 1 — Supplementary Information. [file 41598_2022_22085_MOESM1_ESM.pdf]

Psychotropic drug purchases during the COVID-19 pandemic in Italy and their relationship with mobility restrictions

Francesca Marazzi[1,+], Andrea Piano Mortari[2,7,+], Federico Belotti[2], Giuseppe Carrà[3], Ciro Cattuto[4,5], Joanna Kopinska[6], Daniela Paolotti[4,*], and Vincenzo Atella[2]

[1]University of Rome Tor Vergata, Centre for Economic and International Studies, Rome, 00133, Italy
[2]University of Rome Tor Vergata, Department of Economics and Finance, Rome, 00133, Italy
[3]University of Milano Bicocca, School of Medicine and Surgery, Milan, 20126, Italy
[4]ISI Foundation, Turin, 10126, Italy
[5]University of Turin, Department of Informatics, Turin, 10124, Italy
[6]University of Rome La Sapienza, Department of Social Sciences and Economics, Rome, 00185, Italy
[7]Directorate General for Planning, Ministry of Health, Rome, 00144, Italy
[*]E-mail: daniela.paolotti@gmail.com
[+]These authors contributed equally to this work

ABSTRACT

Table A.1. Drugstores' purchases of anxiolytics and antidepressant in Italy in 2019 and 2020 in DDD

	Drugstore's purchases	Reimbursable prescriptions	% not reimbursed	Pct Var purchases
Anxiolytics (N05B)				
2019	481,098,516	36,687,947	92.37%	-
2020	502,369,453	35,099,829	93.01%	4.42%
Antidepressants (N06A)				
2019	1,126,019,226	964,474,493	14.35%	-
2020	1,153,648,647	986,233,682	14.51%	2.45%

Notes: the amount not reimbursed is either sold via Non Reimbursable prescriptions (NR-Rx), over the counter or kept as pharmacies' stock. The percent variation between 2020 and 2019 is computed as $\frac{2020-2019}{2019} \cdot 100$, for drugstores' purchases only.

Table A.2. Differences in the average monthly purchases between 2020 and 2019

A: Anxiolytics				
	2020	2019	δ	Pct Var
Jan	431,524.70	416,397.58	15,127.12***	3.85%
Feb	379,457.84	354,783.93	24,673.91***	6.51%
Mar	434,725.57	380,593.90	54,131.67***	14.38%
Apr	369,953.39	364,083.32	5,870.07*	1.13%
May	363,331.40	402,864.36	-39,532.96***	-9.50%
Jun	394,027.48	363,391.28	30,636.20***	9.91%
Jul	424,975.44	404,425.66	20,549.78***	5.74%
Aug	313,957.28	308,332.87	5,624.41**	1.36%
Sep	419,192.29	395,721.37	23,470.92***	6.30%
Oct	405,240.57	396,553.90	8,686.67***	2.84%
Nov	376,233.58	365,480.13	10,753.45***	3.12%
Dec	382,422.07	343,619.50	38,802.57***	10.58%
B: Antidepressants				
	2020	2019	δ	Pct Var
Jan	1,009,613.67	989,128.88	20,484.79***	2.17%
Feb	857,378.65	785,791.0	71,587.57***	8.88%
Mar	1,055,734.24	876,379.77	179,354.48***	20.98%
Apr	824,951.24	830,934.79	-5,983.55	-0.73%
May	816,883.63	968,074.50	-151,190.87***	-15.00%
Jun	904,362.65	870,376.60	33,986.05***	4.75%
Jul	984,656.70	954,744.46	29,912.24***	3.30%
Aug	717,513.26	733,559.52	-16,046.26***	-2.02%
Sep	962,778.30	923,405.21	39,373.08***	4.81%
Oct	929,833.88	934,629.80	-4,795.93	0.00%
Nov	865,664.37	860,387.33	5,277.04	1.02%
Dec	852,392.45	796,132.21	56,260.24***	7.77%

Notes: p-values from paired tests run on average drugs purchased at the Province level ,* for p<.05, ** for p<.01, and *** for p<.001.

Figure A.1. Monthly drugstores' purchases and reimbursable prescriptions of anxiolytics and antidepressants, percentage change between 2020 and 2019

(a) Anxiolytics

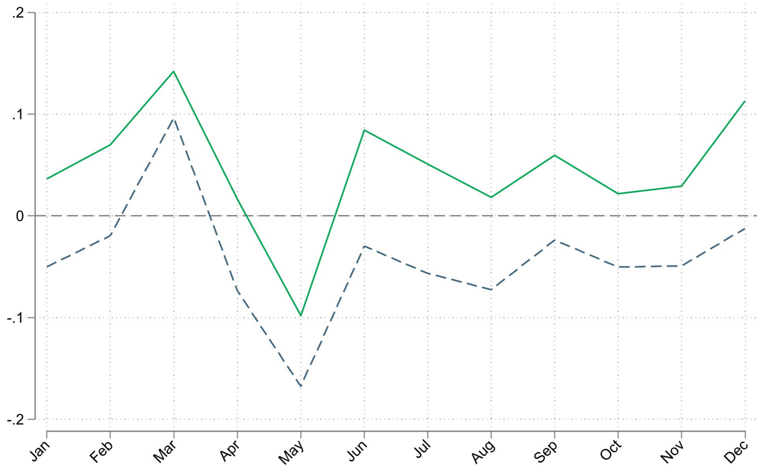

(b) Antidepressants

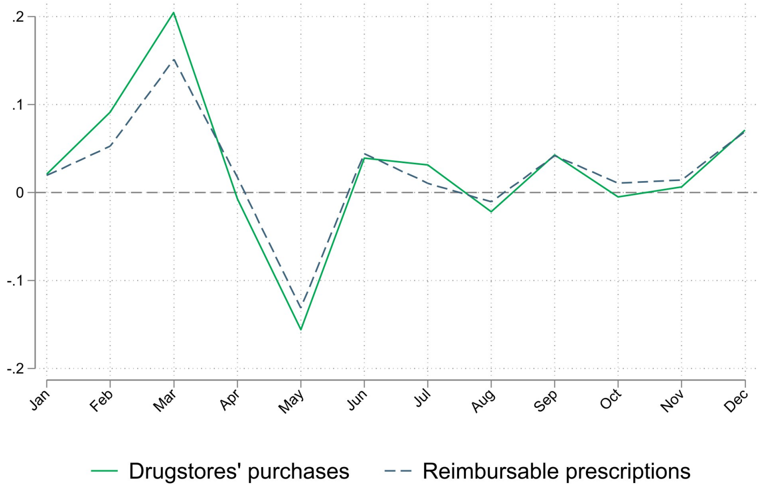

Note: monthly percentage change of drugstores' purchases (green solid line) and reimbursable prescriptions (blue dashed line) of 2020 with respect to the same month in 2019. Anxiolytics (panel A.1a) refers to ATC code N05B and Antidepressants (panel A.1b) to ATC code N06A.
Source: our elaboration of IQVIA data.

Figure A.2. Self-reported solutions for stress, mental health and mental wellbeing (other European countries)

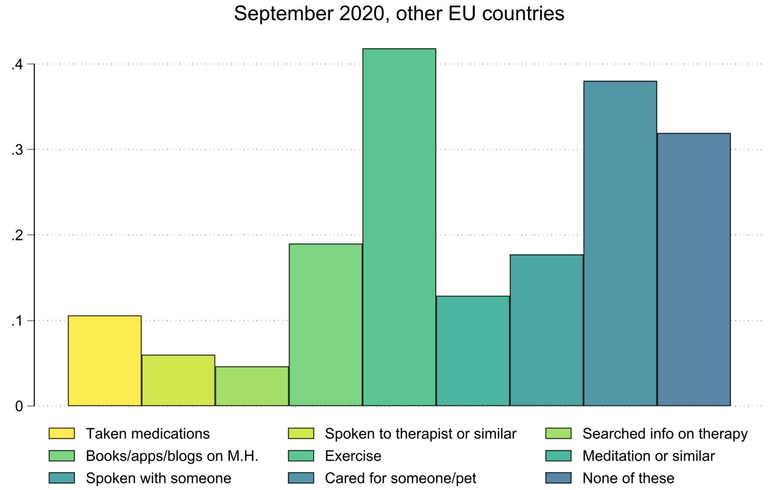

Note: answers to the question: "In the past week have you done any of the following [to improve your stress, mental health or mental wellbeing]? Please tick all that apply." The sample includes all the European countries available, except Italy, i.e. Denmark, Finland, France, Germany, Netherlands, Norway, Spain, Sweden and United Kingdom ($N = 8,328$).
Source: Our elaboration of data provided by Imperial College London YouGov COVID-19 Behaviour Tracker Data Hub
